# Cell Fate Decisions During Breast Cancer Development

**DOI:** 10.3390/jdb4010004

**Published:** 2016-01-22

**Authors:** Kayla Gross, Ania Wronski, Adam Skibinski, Sarah Phillips, Charlotte Kuperwasser

**Affiliations:** 1 Department of Developmental, Molecular and Chemical Biology, Sackler School of Graduate Biomedical Sciences, Tufts University School of Medicine, 136 Harrison Ave., Boston, MA 02111, USA; 2 Raymond and Beverly Sackler Convergence Laboratory, Tufts University School of Medicine, 145 Harrison Ave., Boston, MA 02111, USA; 3 Molecular Oncology Research Institute, Tufts Medical Center, 800 Washington St., Boston, MA 02111, USA

**Keywords:** breast cancer, cell fate, mammary epithelial cells, plasticity, cancer heterogeneity, mammary gland

## Abstract

During the formation of breast cancer, many genes become altered as cells evolve progressively from normal to a pre-malignant to a malignant state of growth. How mutations in genes lead to specific subtypes of human breast cancer is only partially understood. Here we review how initial genetic or epigenetic alterations within mammary epithelial cells (MECs) can alter cell fate decisions and put pre-malignant cells on a path towards cancer development with specific phenotypes. Understanding the early stages of breast cancer initiation and progression and how normal developmental processes are hijacked during transformation has significant implications for improving early detection and prevention of breast cancer. In addition, insights gleaned from this understanding may also be important for developing subtype-specific treatment options.

## 1. Introduction

Over the past three decades, enormous strides have been made in understanding the genetic and biochemical defects in cancer cells that are responsible for deregulated growth and proliferation. Research has revealed critical mutations in many key cellular genes, specifically oncogenes and tumor suppressor genes, within the genomes of a wide variety of human breast cancer cells. During the formation of the majority of breast cancers, these genes become altered as cells evolve progressively from normal to a pre-malignant to a malignant state of growth.

Although these findings have been most illustrative, they fail to inform us about the details of how specific subtypes of human breast cancer begin. A significant number of genes have been catalogued in the genomes of highly advanced breast carcinomas that are detected in the clinic [1]. While this research has revealed the final steps leading up to the appearance of aggressive breast cancers, we have only recently begun to uncover the nature of the early steps of breast cancer development. Here we review how initial genetic or epigenetic alterations in mammary epithelial cells (MECs) can alter cell fate decisions during the earliest steps of neoplastic transformation. We focus on work that has identified the cell-of-origin for various breast cancer subtypes and how cell fate changes in different precursor cells leads to tumors with different phenotypes and behaviors. This new understanding has significant implications for early detection and prevention of breast cancer, as well as possible development of improved therapeutics unique for each tumor subtype. However, in order to prevent breast cancer formation, much remains to be uncovered regarding how the cell-of-origin responds to mutation, stress, DNA damage and aging.

## 2. Breast Cancer and Its Complex Heterogeneity

Breast cancer is a multifaceted disease, consisting of tumors with varying levels of heterogeneity, which includes differences in clinical characteristics, molecular profiles, metastatic behavior, and therapeutic responsiveness. Breast tumors are classified based on the tumor’s morphology and structural organization, with the most common histological type of invasive breast tumor being invasive ductal carcinoma [2,3]. In addition to morphological profiling, immunohistochemical analyses for specific markers—including estrogen receptor (ERα), progesterone receptor (PR), and human epidermal growth factor receptor 2 (HER2/NEU/ERBB2)—have allowed breast tumors to be classified into different subtypes. These categories include: ERα^+^ (ERα^+^/HER2^−^); HER2^+^ (ER^−^/HER2^+^); triple negative (TN; ERα^−^/PR^−^/HER2^−^), and triple positive (ERα^+^/PR^+^/HER2^+^) [2]. These subtypes also have been correlated with trends in overall prognosis and helped identify tumors that may respond favorably to targeted therapies [2]. These general immunohistochemical subtypes also contain heterogeneity within their respective groups. For example, TN breast cancers demonstrate diverse histological patterning and few consistently recurring mutational trends aside from TP53, PTEN, and PICK3CA, the latter concern being a large obstacle for developing targeted therapies [4]. Recently, TN breast cancer has been subcategorized into six subtypes; the majority of those subtypes correlate with a basal-like profile, though some overlap with other molecular subtypes [4]. Though currently less used in clinical settings, molecular profiling, as determined by high-throughput gene expression and hierarchical clustering, is another way breast tumor heterogeneity is defined. This method classifies tumors into several “intrinsic” breast cancer subtypes: luminal-like (also further subdivided into luminal A and luminal B), basal-like, HER2-enriched, and claudin-low [5,6,7].

**Figure 1:**
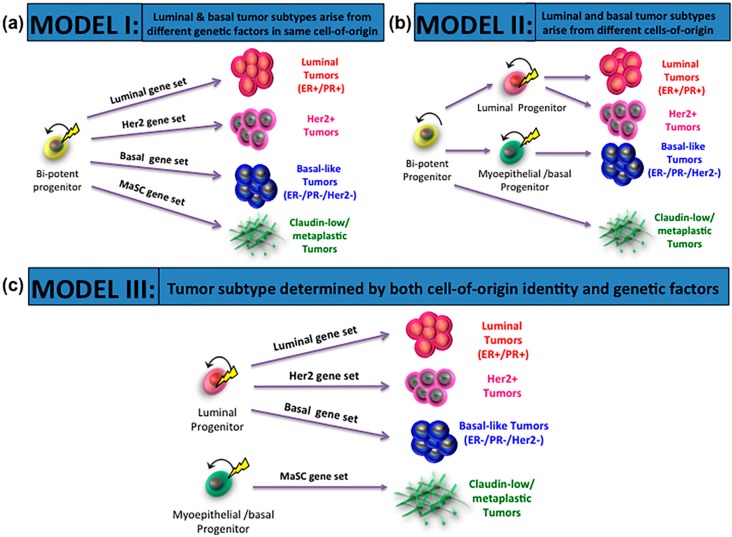
Schematic models of origins of breast cancer heterogeneity (**a**) Model I posits that mutations associated with neoplastic transformation determine subtype; (**b**) Model II posits that the cell-of-origin identity determines subtype during neoplastic transformation; (**c**) Model III posits that both mutation-of-origin and cell-of-origin contribute to the path of disease progression in regards to subtype development.

Two models have emerged that attempt to explain the diverse heterogeneity observed in breast cancer: (1) the “mutation-of-origin” model or (2) the “cell-of-origin” model. The first model posits that all tumor subtypes are derived from a common precursor cell—a bipotent mammary stem cell—but that specific genetic and epigenetic alterations acquired during the process of neoplastic transformation affects that cell’s ability to commit to either the luminal or basal lineage (Figure 1a). Thus, mutations that lock the stem cell’s progeny towards differentiating down the luminal lineage would result in luminal-type tumors, while those that trap stem cells into the basal fate would give rise to basal-like tumors. Based on this assumption, one would expect to observe common and recurring mutations in specific breast cancer subtypes. Indeed, large-scale sequencing efforts have shown that luminal- and basal-like breast cancers exhibit specific and generally non-overlapping mutations. For example, amplification of cyclin D1 and MDM2 or mutations in TBX3 and RUNX1 are preferentially observed in luminal-like breast cancers, while mutations in p53, pRb, BRCA1 and PTEN are commonly found among basal-like tumors [1].

In contrast, the “cell-of-origin” model hypothesizes that a cell’s differentiation program is so tightly encoded it survives the neoplastic transformation process (Figure 1b). Thus, luminal-type tumors would be derived from luminal progenitor cells while basal-type tumors would be derived from basal/myoepithelial progenitor cells. This theory is supported by the fact that molecular profiles of luminal-type tumors share many similarities with that of normal luminal mammary epithelial cells; they express luminal cluster genes (cytokeratins (CKs) *8, 18, 19,* and *7*) as well as luminal differentiation-associated genes (*GATA3*, *EPCAM*, *MUC1*, *CD24,* and *ERα*) [3,5]. Likewise, basal-like tumors closely resemble gene expression programs of their normal basal mammary epithelial counterparts, with expression of basal cluster genes including CKs *5*, *6*, and *17*, as well as *CD49f*, *CD44*, *LAMININ*, and *p63* [3,5]. In addition, experimental support for this model comes from studies where transforming different cell types with the same set of oncogenes gives rise to tumors with different characteristics and phenotypes that are reminiscent of the cell-of-origin [8,9].

In reality however, a third hybrid model has emerged: breast cancer heterogeneity and subtype differences appear to arise from a combination of both the mutation-of-origin and the cell-of-origin (Figure 1c). How these two categorical drivers of breast tumor identity and diversity interact is still being investigated, but recent exciting discoveries have begun to tease apart the convergence of cellular and mutational origins of breast cancer.

## 3. Cells-of-Origin and Impact on Breast Cancer Subtypes

Several complementary and important advances in the field have occurred to identify the cellular origin of breast cancer. First, gene signature sets for the various subsets of normal mammary epithelial cells—including mammary stem cell (MaSC)-enriched, luminal progenitor, mature luminal, and stromal populations—were derived [3,10,11]. These normal gene signatures were subsequently compared to gene expression datasets for the breast cancer molecular subtypes. As hypothesized, the luminal gene signature had the highest degree of overlap with the luminal-like tumor type. Similarly, the stromal gene signature correlated with claudin-low type tumors, a result consistent with the mesenchymal features characteristic of this subtype [10]. The claudin-low transcriptional profile is also similar to the metaplastic CD10^+^ profiles and expresses markers of the epithelial-to-mesenchymal transition (EMT) and cancer stem cells [12]. Remarkably however, the expression signature of basal-like tumors showed a remarkable similarity to the luminal progenitor gene signature [10]. This finding was further supported by immunophenotypic profiling of human breast cancer tissues that revealed that basal-like tumors as well as Her2 tumors were comprised of luminal (EpCAM^+^/CD49f^+^) cells [13]. Therefore, not surprisingly, the gene signature of HER2 tumors does not overlap with any normal mammary epithelial cell type [3,10].

Second, experimental evidence functionally defining the cellular origins of breast cancer was reported [14,15]. Normal luminal and basal mammary epithelial cells were isolated and sorted from reduction mammoplasty tissue and infected using various combinations of transforming oncogenes; these infected cells were immediately implanted into immunocompromised humanized mice to create spontaneous tumors [14,15]. When luminal cells were infected and implanted into mice, they formed both ER^+^ luminal-like tumors and ER^−^ basal-like tumors. In contrast, when basal cells were infected and implanted into mice, rare metaplastic tumors formed, which resembled the claudin-lo subtype. These studies were the first to show that in human breast tissue, cells from the luminal lineage contained precursors to basal-like breast cancer [14,15]. The finding that the cell of origin to luminal and basal tumors are within the luminal lineage has also been supported in mouse tumor models. MMTV-PyMT and MMTV-Neu mice develop tumors of the luminal-like subtype, while Etv6-NTRK3 mice form basal-like mammary tumors. In all three of these models, tumors were found to originate from CD61^+^ alveolar progenitor-enriched luminal cells [16]. Using lineage-specific drivers of cancer, targeted loss of *BRCA1* in luminal cells, but not basal cells, produced basal-like tumors [17]. A more recent study using targeted deletion of Brca2, Pten and p53 in mice also showed that when these genes were lost in basal MECs, the same tumor phenotype always emerged—one that resembled claudin-low tumors. In contrast, depending on the initiating genetic lesion in luminal MECs, tumor-initiating cells from this lineage gave rise to basal-like, luminal-like, and normal-like tumors [18]. This important study not only demonstrated that multiple mammary tumor subtypes can arise from the same cell-of-origin pool, but also that molecular subtype cannot be used to infer tumor cell-of-origin identity. This study also illustrates how both the mutation-of-origin and the order in which the mutations occur influences the path of neoplastic transformation.

## 4. Mutations-of-Origin and Impact on Breast Cancer Subtypes

If luminal cells, and most likely luminal progenitor cells, are the precursors to the most common forms of breast cancer, then it stands to reason that genetic mutations contribute strongly to the fate of luminal cells during cancer formation. A classic example of this has been shown in women who inherit mutations in the Breast Cancer Associated 1 gene (*BRCA1*). Women with mutations in *BRCA1* preferentially develop basal-like tumors, and only recently has exploration into the underlying mechanism for this correlation begun to yield results. Gene expression analysis of *BRCA1*-associated breast cancer strongly correlates with the gene expression signature of luminal progenitor cells; *BRCA1*-mutation carriers in this study appeared to contain an expanded luminal progenitor population [10]. However, a different study also examining cells from *BRCA1*-mutation carriers failed to observe this expansion; rather they found an expansion of the basal progenitor population [14]. Despite the increased number of basal cells in *BRCA1*-mutation carriers, this study showed that the luminal cells, and not the basal cells, were the origins of basal-like breast cancer [14]. When probed more deeply, it was found that luminal progenitor cells from *BRCA1*-mutation carriers exhibited a defect in differentiation, one in which progenitor cells could not give rise to mature cells. Instead, progenitor cells gave rise to luminal cells that expressed basal markers [14]. This revealed that an early genetic mutation, even prior to cancer formation, could alter the fate of the cell-of-origin so that it was predisposed for forming tumors of a specific subtype.

Mouse tumor models have also supported the notion that the fate of luminal progenitor cells can be affected by transforming oncogenes. In mice harboring mutations in the PI3K-pathway effector *PIK3CA,* mammary tumors arise with various characteristics including those of basal-like breast cancer. Interestingly, luminal cells in these mice exhibit an altered fate and de-differentiate into multipotent stem-like cells [19,20,21,22]. Viral introduction of tagged oncogenes (ErbB2, PyMT) into luminal cells *in vivo* can also influence cell fate during neoplastic transformation [21]. In this model, a small percentage of luminal cells demonstrated basal marker (K5^+^) expression following oncogene transduction indicating that luminal cells were adopting a basal fate [23]. These findings all support the concept that gene mutations do affect the fate of the cell-of-origin, directing tumor phenotype during neoplastic transformation.

## 5. Mechanisms Affecting Cell Fate Changes

If luminal-committed progenitor cells are the precursors for the most common forms of breast cancer, including basal-like, how is it that lineage-restricted cells can adopt phenotypes of a different lineage? Can other precursor sources also undergo similar cell fate switching mechanisms that contribute to subtype development as well? De-differentiation and trans-differentiation both have been established as viable options for cell fate switching in the mammary gland, though the exact mechanisms have only begun to be elucidated.

In the case of *BRCA1*-mutation carriers, aberrant expression of the transcription factor Slug was shown to be the culprit for cell fate switching. Mutation in *BRCA1* was reported to promote stability of the Slug protein [14]. Slug in turn could enhance the expression of basal and stem-related genes as well as associate with and recruit the lysine-specific histone demethylase LSD1 to the promoters of luminal genes to prevent their expression [24]. This role as a cell fate regulator was confirmed *in vivo* using a functional Slug knockout model, which revealed the expression of luminal genes in basal cells, solidifying the importance of Slug as a determinant of basal cell identity [24].

In other contexts, the mechanisms that induce cell state transitions or promote lineage switching are less clear. Whether the switch in cell fate occurs though trans-differentiation or de-differentiation into a more unrestricted primitive state is not known. Examples from other tissues suggest that both can occur and that the cell fate decision may be cell type- or mutation-specific. In the pancreas, trans-differentiation of acinar cells into duct-like cells after *Notch-* and *Kras-*drive reprogramming eventually leads to ductal intraepithelial neoplasia [25,26]. However, in basal cell carcinoma, interfollicular epidermal cells serve as the cells-of-origin, and are reprogrammed back into a more progenitor-like state before progressing towards carcinoma, possibly by transitioning through an EMT phase [27,28,29]. Likewise in the intestine, while LGR5^+^ or BMI1-expressing stem cells do give rise to adenomas, villus cells with abnormal Wnt signaling are able to de-differentiate and exhibit tumor-forming capacity [28,29,30,31,32]. These studies show that identity changes within specific cell populations can occur through multiple routes during tumor formation and may be dependent on the cells-of-origin as well as the early oncogenic events during neoplastic transformation.

In the mammary gland, transplantation can promote lineage switching by inducing de-differentiation of restricted progenitors into bipotent stem cells [33,34,35]. Interestingly, Slug appears to control this multipotent fate potential of MECs during transplantation but also during tumorigenesis. MECs from mice expressing a mutant of Slug that lacks transcriptional activity are unable to unlock stem cell programs needed to regenerate mammary tissue following transplantation or to form tumors driven by Myc [24,36].

While it has been shown that lineage switching of MECs can occur through activating multipotency, there is also evidence demonstrating that luminal cells can directly trans-differentiate into basal cells without having to transition through a stem cell intermediate. Expression of the hippo transducer TAZ was sufficient to promote basal cell fate through regulating expression of basal lineage genes in concert with the SWI/SNF chromatin-remodeling complex [37]. Interestingly, elevated TAZ expression and gene amplification is associated with a basal-like tumor phenotype and correlates with poor survival, possibly suggesting that selection for a basal MEC lineage regulator during tumorigenesis may encourage a shift towards a more basal-like cell state [1,37].

Another regulator of basal cell identity is LBH (limb bud and heart development), which is a Wnt-controlled transcriptional co-factor expressed in a rare population of stem-like cells that reside within the mammary basal lineage [38]. It was shown to control basal cell identity by inducing ΔNp63 gene expression while also repressing ERα gene expression. Loss of LBH impeded normal mammary gland development and consequently its expression is crucial for mammary stem cell maintenance and function, as well as lineage specification [38]. As LBH is overexpressed in basal-like breast cancer, as suggested above for Taz, its selective activation during neoplastic transformation may influence the path of disease progression in relation to cell fate and thus ultimately affect breast tumor subtype determination [39,40].

Cell fate switching has also been shown not only to occur in the luminal cell compartment of the mammary gland, but in the basal cell compartment as well. Mice lacking the transcriptional repressor *Ovol2* exhibit disrupted mammary morphogenesis because loss of Ovol2 in basal cells causes them to become non-epithelial cell types, including fibroblast and muscle [41]. As Ovol2 is significantly downregulated in claudin-low-type breast cancers, this suggests that cell identity-related disruptions within the basal cell compartment may also contribute to subtype specification during breast cancer development [41]. In addition, this fortifies the notion that basal cells can serve as the precursors to claudin-low/metaplastic tumors that exhibit non-epithelial features.

While transcription factors are the major orchestrators of directing and maintaining cell identity programs, epigenetic mechanisms—such as DNA methylation and histone modifications—enable activation or repression of transcription factors and thus are also master controllers of such transcriptional programs. In the breast, the relationship between gene expression and DNA methylation was shown in sorted hMECs [42]. Sorted CD44^+^ progenitor cells demonstrated hypomethylation on promoters of genes related to pluripotency and self-renewal functions. Many of these genes were targets of the polycomb repressive complex 2 (PCR2) component Suz12 and were also highly expressed in the CD44^+^ progenitor-enriched cell population. In contrast, more differentiated CD24^+^ cell populations exhibited increased DNA methylation at promoters of Suz12 target genes, suggesting that differentiation may induce epigenetic marks that block activation of stem programs and contribute to directing cell identity [42]. A more recent study also established a similar but more comprehensive epigenetic/transcriptional map by comparing the epigenetic landscape of cells with their transcriptome profiles. Using diverse RNA, DNA, and chromatin integrative analyses, Gascard *et al.* identified and defined various unmethylated regions (UMR) in regulatory elements of genes that direct cell type [43]. When comparing these UMRs in luminal *versus* basal cells, there were almost twice as many UMRs present in the proximal regulatory regions of various transcription factor genes in the luminal cells and that these cell-type specific UMRs correlated with increased gene expression of these transcription factors [43].

How DNA methylation fate is marked in these different cell populations is beginning to be elucidated. The DNA methyltransferase DNMT1 was shown to be necessary for MaSC maintenance; its loss *in vivo* resulted in decreased basal (CD24^+^/CD49f^hi^) and luminal progenitor (CD24^+^/CD49f^lo^CD61^+^) populations, lowered their regenerative and repopulating ability, and hindered tumorigenesis [44]. Uncovering the ability of this epigenetic regulator to impact both normal mammary function and tumor progression is a first step in beginning to understand the mechanism behind DNA methylation influences on mammary cell fate and how that may impact tumorigenic potential.

Histone modifications are another class of epigenetic marks that are critical regulators of MEC identity and fate switching. Global histone methylation profiles of sorted mouse MEC populations were shown to correlate with cell type-specific gene expression profiles. For example, genes upregulated in luminal progenitor (CD24^+^/CD49f^lo^/CD61^+^) *versus* basal/MaSC (CD24^−^/CD49f^hi^) cells had increased H3K4me3 and decreased H3K27me3 marks, and vice versa for downregulated genes. Similar correlative trends between gene expression and histone methylation patterns were observed when comparing the luminal progenitor and mature luminal (CD24^+^/CD49f^lo^/CD61^−^) populations [45]. There was also an interesting association between H3K27me3 marks during hormone-dependent changes during pregnancy; H3K27me3 patterns correlated with gene expression patterns. This trend was linked with EZH2-dependent maintenance of H3K27me3 marks, as loss of EZH2 decreased the presence of this mark [45]. Another regulator of histone modifications, the histone methylation sensor Pygo2, was shown to be crucial for proper stem/progenitor maintenance in the mammary gland, as knockout of Pygo2 in a mouse model exhibited a decrease in basal/MaSC and luminal progenitor populations [46]. Additionally, in these mice, the basal/MaSC population showed a luminal differentiation bias, suggesting that loss of this epigenetic regulator was shifting basal cell identity towards a more luminal one. Mechanistically, the increase in luminal fate of the Pygo2-deficient basal/MaSC was reported to occur through Pygo2 normally maintaining a bivalent (H3K4me3/H3K27me3) mark at the Notch3 gene promoter and thus its absence allowed activation of Notch signaling and its related luminal differentiation programs [46]. These initial characterizations of cell type-specific and developmental stage-specific trends in histone methylation further emphasize the importance of epigenetics and the molecular regulators of those marks in regulating mammary cell identity and function.

One characteristic of histone modifications that is particularly crucial in cell fate decisions and how they may be important during malignant transformation are bivalent chromatin marks. Changes in bivalency may shift cell identity into one lineage or another and may therefore affect tumor phenotype. Putative bivalent domains are present in various gene subsets in both luminal and progenitor cell populations, demonstrating that MECs in either lineage may be poised for lineage switching, highlighting the innate plasticity of epithelial cells in the mammary gland [47]. In cancer, bivalent chromatin marks were shown to be important in determining cancer stem cell (CSC) fate. The EMT transcription factor Zeb1 has bivalent chromatin marks at its gene promoter in non-CSCs of basal breast cancer tissue yet upon appropriate signaling can shift to allow for active transcription of Zeb1, thereby enhancing cell plasticity and activating CSC programs [48]. Although the plasticity of bivalency was observed in cancer cells, it is plausible that normal progenitor cells might contain bivalent marks in key transcription factors making them poised for cell state changes. More work is needed to fully understand how the epigenetic landscape of normal cells can influence cell fate and contribute to cancer.

Taken together, regulators of MEC identity are crucial to cell fate determination and switching, and manipulation of MEC lineage regulators can affect the ability of a progenitor cell to make normal cell fate decisions. Additionally, abnormal versions or expression of these regulators during cancer initiation seem to impact tumor phenotype. Identifying additional epigenetic and transcriptional regulators of mammary epithelial cell fate and linage transitioning will provide deeper insight into the complex regulation of differentiation and also will define important molecular drivers of tumor phenotype.

## 6. Exploiting Cell Fate Changes During Development

As initial genetic mutations can alter the fate of tumor initiating/precursor cells, thereby dictating tumor phenotype, it is also plausible that naturally occurring cell fate changes during development might also be co-opted to drive tumor phenotype. For example, pregnancy is associated with acute expansion of the mammary epithelium, particularly the luminal population. Various stem/progenitor populations have been identified prior to and after pregnancy, and these primitive bipotent cells demonstrate varying longevities and contributions to gland restructuring [49,50,51,52,53,54]. Given that parity, while protective in the long term, increases short-term breast cancer risk, it is possible that the pregnancy-related changes to luminal MECs primes the gland to be more receptive to cell state transitioning and might be exploited and cooperate with early drivers of malignancy that are associated with pregnancy [3,55].

As another example, the actions of the steroid hormones progesterone and estrogen through the actions of their receptors might play a role in cell fate decision upon transformation. Progesterone exhibits different modes of action in luminal and basal compartments during development. In luminal cells, progesterone increases Rankl expression, which in turn promotes the proliferation of adjacent luminal epithelial cells [56]. Progesterone also can induce the expression of growth hormone, resulting in increased progenitor activity when measured *in vitro* [57]. In basal cells, progesterone stimulates Wnt signaling to promote expansion of the basal progenitor cells as well as induce basal cell proliferation [58,59,60]. Thus, it is possible that progesterone might increase the number of potential cancer precursor cells, which might be targets for mutation and subsequent transformation. Consistent with this, large epidemiological studies have revealed that women who were on hormone replacement therapy containing estrogen and progesterone, but not estrogen alone, had a significantly higher incicence of breast cancer [61,62,63]. The mechanism for this remains unclear, but it is plausible that it could be due in part, to the effects of progesterone on cell fate changes and progenitor cell biology.

Historically, ERα expression has been thought to be limited to mature luminal cells within the developing mammary gland as estrogen is hypothesized to function through an indirect proliferative response [64]. However, rare populations of ERα+ cells have been identified in populations with progenitor-like characteristics [65]. The independent function of ER signaling has been difficult to ascertain as ER and PR are commonly co-expressed and estrogen can increase the expression of PR [64,66]. Regardless, ER is a powerful mediator of a number of transcription factors, which in turn regular cell fate within the mammary gland. For example, both PR and ER regulate Stat5a, which is necessary for ductal branching and tumorigenesis [67].

Aging is another naturally occurring stage where cell fate changes may contribute to breast cancer subtype proclivity. Unlike in young women where basal-like breast cancers are found more frequently, luminal-like hormone-positive breast cancers are the major tumor subtype observed in older women [68]. The underlying mechanism behind this tumor subtype bias associated with aging is unclear; however, evidence has emerged that indicates that luminal cells in breast tissues of older women exhibit altered phenotypes and behaviors. Firstly, the number of MECs expressing ERα increase, as well as a concurrent trend towards increased co-expression with the proliferation marker Ki67, suggesting shifts in differentiation and proliferative potential of luminal cells [69,70,71,72]. Second, disease-free breast tissue of older women contains an abnormal luminal progenitor population with impaired differentiation capacity, as its progeny gives rise to luminal cells expressing basal markers [73,74]. Third, while luminal progenitor cells contain abnormally short telomeres independent of age compared to other populations of normal mammary epithelial cells, a decline in telomerase activity—often associated with activation of differentiation programs—was found with increasing age [75,76]. Taken together, these findings suggest that aging affects the ability of the luminal lineage to carry out normal differentiation and stem cell function, thus resulting in defective capacity of these cells to properly maintain and/or shift identity.

Whether or not these changes contribute to the luminal subtype bias observed during aging has yet to be established. However, there is some emerging evidence that might support this connection. Age-related gene expression signatures from disease-free pre- or perimenopausal breast tissue of young women (40 years) correlated with higher grade and aggressive breast tumors, while gene expression signatures from disease-free pre- or perimenopausal breast tissue of older women (>40 years) correlated with lower grade and less aggressive breast tumors [77]. This suggests that aging may alter the transcriptional landscape of the breast, potentially in a way that influences breast tumor development. Interestingly, this same study also tested for gene expression changes based on menopausal status alone and found no significant trends separating gene expression profiles of pre/peri and post-menopausal tissue. Despite this, the hormonal changes and their downstream effects that occur with menopause need to be better characterized to truly determine what effects this important age-related shift in endocrine signaling might have on the breast and its cell populations and how it interacts with other aging-related processes.

Epigenetic changes have also been reported to contribute to aging-related changes to the breast. In normal human breast tissue, older women exhibited increased DNA methylation of CpG islands and polycomb group protein target gene (PCGT) enrichment [78]. Intriguingly, a subset of these CpG gene loci were also hypermethylated in tumors, with some showing a specificity for ER-positive tumors—the subtype that is more prevalent in older women—thus, drawing a connection between epigenetic changes that occur during aging and those that occur during tumor development [78]. Further investigation into the mechanisms by which epigenetic regulation influences cell identity within the mammary gland is needed. In particular, understanding how aging induces shifts in the epigenome and subsequent functional changes that contribute to altered cell fate decisions will be needed to determine how this can influence the heterogeneity of breast tumors.

Collectively, these observations put forth the possibility that as the breast ages, changes in tumor initiating/precursor cells might alter their differentiation potential and cell fate thereby priming the epigenetic landscape to impact tumor phenotype following neoplastic transformation. Additional studies and experimental evidence using models of aging will be necessary to directly support this hypothesis.

## 7. Conclusions

As discussed above, recent work has endeavored to understand the genetic and cellular changes to mammary epithelial cells that occur during mammary development and how they may be utilized during neoplastic transformation to promote breast tumor heterogeneity. Understanding the mechanisms that drive cell fate switching during the transformation process may be the key to improved therapies, and even disease prevention. It is too early to know whether this understanding will impact treatment but it may shed insights into how we may be able to prevent breast cancer from developing or progressing in the future.
